# Association between Inguinal Hernia and Arterial Disease: A Preliminary Report

**DOI:** 10.3390/biology10080736

**Published:** 2021-08-01

**Authors:** Raffaele Serra, Umberto Marcello Bracale, Rosy Conforto, Arturo Roncone, Nicola Ielapi, Ashour Michael, Maurizio Sodo, Maria Donata Di Taranto, Pasquale Mastroroberto, Giuseppe Filiberto Serraino, Michele Provenzano, Michele Andreucci

**Affiliations:** 1 Interuniversity Center of Phlebolymphology (CIFL), “Magna Graecia” University of Catanzaro, 88100 Catanzaro, Italy; rosyconforto@outlook.com; 2 Department of Medical and Surgical Sciences, University Magna Graecia of Catanzaro, 88100 Catanzaro, Italy; michiprov@hotmail.it; 3 Department of Public Health, University of Naples “Federico II”, 80100 Naples, Italy; umbertomarcello.bracale@unina.it (U.M.B.); sodo@unina.it (M.S.); 4 Department Surgery, Hospital of Soverato, 88068 Soverato, Italy; arturo.roncone@gmail.com; 5 Department of Public Health and Infectious Disease, “Sapienza” University of Rome, 00185 Rome, Italy; nicola.ielapi@uniroma1.it; 6 Department of Health Sciences, “Magna Graecia” University, 88100 Catanzaro, Italy; ashourmichael@yahoo.com (A.M.); andreucci@unicz.it (M.A.); 7 Department of Molecular Medicine and Medical Biotechnology, University Federico II of Naples, 80100 Naples, Italy; mariadonata.ditaranto@unina.it; 8 Department of Experimental and Clinical Medicine, University of Catanzaro, 88100 Catanzaro, Italy; mastroroberto@unicz.it (P.M.); serraino@unicz.it (G.F.S.)

**Keywords:** arterial disease, inguinal hernia, vascular, carotid, aneurism, peripheral artery disease, metalloproteinases

## Abstract

**Simple Summary:**

While the association between venous disease and inguinal hernia has been well demonstrated, there is less evidence concerning the association between arterial diseases (AD), such as carotid stenosis, peripheral artery disease and abdominal aortic aneurysms, and inguinal hernia. We surprisingly found that the prevalence of AD is large, being higher than 40% in our study cohort. Moreover, patients with AD as compared to those without AD are characterized by additional other comorbidities such as greater albuminuria, higher frequency of a smoking habit and older age. Hence, we provided a characterization of patients with inguinal hernia with respect to concomitant presence of AD.

**Abstract:**

Background: Inguinal hernia (IH) is a major problem in general surgery and its prevalence is increasing. The presence of hernias has been associated with a wide spectrum of venous diseases, with the involvement of imbalances in collagen and extracellular matrix deposition and metalloproteinases dysfunction. We aimed to evaluate whether the association between IH and vascular diseases is also present with respect to arterial diseases. Methods: We designed a cross-sectional observational study enrolling consecutive patients undergoing surgical repair of IH. Arterial diseases (AD) considered were carotid stenosis, peripheral artery disease and abdominal aortic aneurysms. Results: Study population consisted of 70 patients. Mean age was 63.2 ± 4.7 years. Prevalence of AD was 42.9% in the whole cohort. AD patients were older (*p* = 0.015), and more frequently had hypertension (*p* = 0.001) and active smoking habits (*p* = 0.001) than the no-AD group. Albumin-to-creatinine ratio (ACR) was higher in AD than in no-AD patients (*p* < 0.001). At multivariable analysis, increased ACR (odds ratio, OR: 1.14, *p* < 0.001), old age (OR: 1.25, *p* = 0.001) and a smoking habit (OR: 3.20, *p* = 0.001) were significant correlates for the presence of AD. Conclusions: Prevalence of AD in patients with IH is non-negligible. Old age, a smoking habit and an abnormal excretion of urine albumin are associated with the presence of AD in these patients. Future studies are needed to gain more insights into the pathogenic mechanisms underlying this association, exploring also the specific role of metalloproteinases.

## 1. Introduction

Inguinal hernia (IH) is a common clinical condition, and its incidence seems to increase with age and with the male sex. As such, IH repair is one of the most performed operations in general surgery. There are several etiologic factors for the development of IH, such as extracellular matrix (ECM) imbalance, which contributes to the reduction in strength of the abdominal muscle wall [1,2]. In particular, the disequilibrium between the quality, quantity and arrangement of collagen and elastin composition of the transversalis fascia was considered to be one of the main etiopathogenetic factors of IH [1]. Previous studies have shown an association between some vascular diseases, such as a varicocele and chronic venous disease (CVD), and the onset of inguinal hernia, suggesting a common pathogenetic denominator in ECM alterations due to an imbalance of matrix metalloproteinases (MMPs) [3]. While further studies are needed to confirm the aforementioned relationship, nonetheless there is enough evidence to warrant an investigation of the association between IH and arterial disease (AD), as this condition is also related to ECM alterations of the vessel wall. Arterial disease (AD) is any condition that affects the arteries, and includes conditions such as carotid stenosis, aneurysms and peripheral artery disease (PAD), which may severely impact the national health systems in terms of morbidity, mortality and quality of life [4]. Carotid stenosis, a narrowing of the carotid arteries, is the highest risk factor for stroke with overall prevalence being higher in men (3.8%) than in women (2.7%) [5,6]. It can be clinically asymptomatic or symptomatic [7]. Aortic aneurysms (AA) have an overall prevalence of 4.8%, which is not negligible given the high risk of rupture, a potentially fatal event [8]. The aorta, among all the arteries, is the vessel most frequently affected by aneurysmal disease, with 80% of cases occurring in the abdominal tract and 20% in the thoracic portion [9]. Peripheral artery disease (PAD) is a slowly progressive steno-obstructive disease of the arterial circulation responsible for the vascularization of the lower extremities, causing reduced blood flow, which can be accompanied by severe symptoms. It occurs in 4–12% of those in the 55–70 age group and its prevalence increases with age. Other risk factors are represented by atherosclerosis, a smoking habit, hypertension, lipid alteration, diabetes and rheological blood properties [10]. The aim of this observational study was to investigate the association of IH with AD, in particular carotid stenosis, abdominal aortic aneurysm and PAD. Such a research question may help clinicians to better investigate AD, even when in an asymptomatic phase, in individual patients with IH as well as increase preventive tools for IH in patients with detected AD. 

## 2. Materials and Methods

This is a cross-sectional observational study enrolling consecutive patients who underwent inguinal hernia surgical repair from 1 January 2019 to 31 December 2020 at the General Surgery Unit of “Basso Ionio” Hospital of Soverato (located in Calabria, South Italy). The study was approved by the Institutional Review Board of the Interuniversity Center of Phlebolymphology (CIFL). The International Research and Educational Program in Clinical and Experimental Biotechnology (approval number: ER.ALL.2018.30.A) and all patients gave written informed consent. The protocol was properly registered at a public trials’ registry, www.clinicaltrial.gov, accessed on 29 July 2021 (NCT04428138). Patients were included if they were aged >18 years and had received an indication for surgical intervention. At hospital admission, the possible concomitant presence of the arterial pathology of interest was derived from the medical history reported by the patient or through the file attached by the general practitioner or by telephone interview. Arterial pathologies collected included carotid stenosis, abdominal AA (AAA) and PAD. Carotid stenosis was diagnosed with the use of an imaging technique, such as a doppler ultrasound of the neck, CT angiogram (CTA) of the neck, magnetic resonance angiography (MRA) or cerebral angiogram. The imaging technique chosen was done so on the basis of the clinical condition of patients. CT angiogram was executed in case of diagnostic uncertainty. Magnetic resonance angiography was preferred in the concomitant presence of diagnostic uncertainty and moderate–severe kidney function impairment (estimated glomerular filtration rate below 60 mL/min/1.73 m^2^) to avoid contrast-induced acute kidney injury. Cerebral angiogram was considered for patients who underwent radiotherapy for head–neck neoplasms that altered results of non-invasive imaging. The percentage of stenosis was also measured. The aneurysms were included if a localized dilation of abdominal aorta greater than 50% of the normal diameter was detected [11,12] The extension in diameter (in centimeters) of AAA was recorded. PAD was detected by both ultrasound and clinical examination and was classified into six stages (Rutherford classification) according to the severity of disease: stage 1 (mild intermittent claudication), stage 2 (moderate intermittent claudication), stage 3 (severe intermittent claudication), stage 4 (rest pain), stage 5 (minor tissue loss) and stage 6 (major tissue loss) [13]. The main demographic and clinical characteristics of the study patients, laboratory parameters and comorbidities were collected during the study visit. Patients were also asked to collect a urine morning void for assessing the urine albumin excretion, measured as albumin-to-creatinine ratio (ACR).

### Statistical Analysis

Sample size was calculated considering mean difference between AD and no-AD groups. A sample size of 35 patients per group achieves 80% power to detect a mean difference of 0.5 standard deviation in terms of mean difference (medium effect size), with a significance level (alpha) of 0.05 using a two-sided independent sample t test. Such a stringent cut-off was selected due to the small sample size. *p* values have been adjusted by means of the Bonferroni method. Continuous variables were reported as either mean ± standard deviation (SD) or median and interquartile range (IQR) based on their distribution. Comparison between arterial disease categories was assessed by one-way ANOVA or Kruskal–Wallis test. Categorical variables were analyzed using the chi-square test. Correlations between the main continuous variables were assessed by means of x–y plots, and r coefficients were computed through the Spearman rank test. For the model building process, univariate analysis testing the association between the main clinical variables and arterial disease, considered as the presence of one or more diseases among carotid stenosis, AAA and PAD, was assessed by means of logistic regression analysis. A backward variable selection method with an elimination criterion of *p* < 0.05 was performed with a multivariate logistic regression model with presence of artery disease included as a dependent variable. Multicollinearity was assessed with variance inflation factors (VIF), which is a measure of the degree to which a single predictor variable can be expressed as a linear combination of the remaining predictor variables; values greater than 10 were considered to be a cause for concern [14]. Due to its skewed distribution, ACR was log-transformed before its inclusion in the multivariable model.

## 3. Results

Our observational analysis consisted of 70 consecutive patients with IH. The overall cohort was characterized by a high cardiovascular risk as shown by the high prevalence of hypertension (62.5%), history of previous cardiovascular disease (including myocardial infarction, stroke, chronic heart failure and arrythmias, with an overall prevalence of 36.7%), current smokers (42.1%) and type II diabetes (25.2%). Median levels of urine albumin (ACR) were 141 mg/g (range 102–215 mg/g). The overall prevalence of AD was 30 out of 70 patients (42.9%). Distribution of AD consisted of 11 patients with carotid stenosis, 14 with AAA and 5 with PAD (Table 1). 

Among patients with carotid stenosis, one underwent diagnostic cerebral angiogram because of previous radiotherapy on the neck region, six underwent doppler ultrasound, two underwent CTA and two underwent MRA. Patients with AD were older, on average, compared to those without AD (*p* = 0.015). Furthermore, the prevalence of hypertension and a smoking habit were higher in patients with carotid stenosis, AAA and PAD than the no-AD group (*p* = 0.001 and *p* = 0.001, respectively). Urine albumin was significantly higher in patients with AD (particularly in AAA and PAD subgroups) compared with the no-AD category (*p* < 0.001). Mean eGFR levels were significantly lower in patients with AD as compared to those with no-AD (*p* = 0.030). No significant differences were found for gender, type II diabetes, body mass index and LDL cholesterol levels among AD categories. Specifically, for the three types of AD, the mean percentage of carotid stenosis was 57.3%, and AAA extension in diameter was 5 ± 1 cm on average (Table 2). 

**Figure 1:**
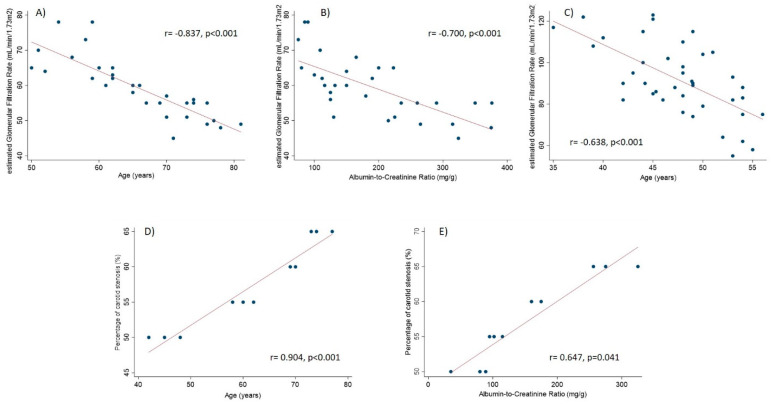
Correlations between the key study variables. Panels **A** and **B** refer to AD group. Panel **C** refers to no-AD group. Panels **D** and **E** refer to subgroup with carotid stenosis.

Two out of five patients with PAD were classified as Rutherford stage 1, whereas one patient was classified as stage 2, one as stage 3 and one as stage 4. When testing correlations between the key continuous variables, we found that age was inversely correlated with eGFR both in AD (r = −0.837, *p* < 0.001) and no-AD (r = −0.638, *p* < 0.001) groups (Figure 1). Estimated glomerular filtration rate was also inversely correlated with ACR in the AD group (r = −0.700, *p* < 0.001). In the sub-group (of AD) of carotid stenosis, the percentage of carotid stenosis was significantly positively correlated with ACR (r = 0.647, *p* = 0.041) and age (r = 0.904, *p* < 0.001). Significant correlations are depicted in Figure 1.

At multivariable logistic regression analysis, after the stringent stepwise selection, older age (*p* = 0.001), current smokers (*p* = 0.001) and ACR (*p* < 0.001) were found as independent correlates for the presence of AD (Table 3).

## 4. Discussion

### 4.1. The Clinical Burden of Inguinal Hernia

Inguinal hernia is one of the most common conditions that requires surgery, affecting one in three male subjects [15]. With respect to age, the incidence of IH has a bimodal distribution. It is, indeed, higher in childhood and in the elderly [16]. Surgical repair of IH is considered the gold standard because it leads to a definitive solution of this problem. However, it is not free from complication, since the risk of hernia recurrence, chronic discomfort and chronic pain is not trivial [15]. Hence, a better comprehension of the pathogenesis of hernia and the risk factors that forecast its development and worsening would help clinicians to improve the management of high-risk patients, in an attempt to reduce the overall cases that need surgical treatment or to expand the therapeutic strategies as well [17,18]. In this context, the presence of hernias has been associated with the presence of vascular venous diseases [19].

### 4.2. Association between Inguinal Hernia and Vascular Diseases

In particular, it has been demonstrated that patients with a varicocele are at increased risk for developing IH and CVD over time, thus implying a strict pathogenic link between these three conditions [20]. An interesting hypothesis, that has been claimed to explain such an association, is the imbalance of the extracellular matrix (ECM), a mechanism that is involved in the development of both hernias and vascular diseases. In fact, increased levels of MMP, particularly MMP-9, have been found in patients with multiple venous diseases as well as hernias [3,21,22]. 

### 4.3. Principal Findings of the Present Study

With the present observational study, we extended the analysis to AD. To our knowledge, this is the first study thus far to assess the association between IH and a wide spectrum of AD, encompassing carotid stenosis, AAA and PAD. The first important finding of our study was the high prevalence of arterial disease in patients who underwent surgical repair for inguinal hernia. In fact, we found that 43% patients in our cohort suffered from AD. The second finding was that the presence of AD in patients with hernias was independently associated with the presence of increased ACR. The increased excretion of albumin (or total protein) with urine is a well-recognized marker of kidney damage [23]. But, even more importantly, the increase in albuminuria in the general population and high-risk patients has been associated with an increased risk for developing cardiovascular events including AAA, PAD and atherosclerotic disease [24,25,26,27]. Matsushita and colleagues showed, in a large meta-analysis, that the predictive ability of ACR for cardiovascular events was equal or even higher than that exerted by traditional risk factors such as blood pressure or LDL cholesterol levels [28]. A novel finding from our work is that we found a significant association between urine albumin and AD, specifically in patients with hernias. Such a link is also intimated by the positive correlation between ACR and the percentage of carotid stenosis we found in our cohort. Additionally, albeit not reaching statistical significance in the multivariable model, we also found a potential role of eGFR in the association with AD in our cohort. In fact, eGFR was significantly associated with ACR being lower for higher levels of ACR. This suggests that eGFR should be monitored in combination with ACR in this setting of patients. An inverse association between eGFR and ACR has been previously shown and it has been well demonstrated that both these kidney measures, eGFR and ACR, are able to predict cardiovascular risk in the general population and high-risk patients [28,29]. 

### 4.4. Hypotheses Generated

We may hypothesize that a common pathogenetic mechanism is shared among AD, inguinal hernia and kidney damage. One of them is likely represented by alterations in the ECM. Increased levels of MMP-9 were found to be associated with higher albuminuria levels in a number of kidney diseases in humans, namely glomerulonephritis and diabetic nephropathy [30,31,32]. At the same time, MMP-2, neutrophil gelatinase-associated lipocalin (NGAL) and MMP-9 are expressed in the aneurysm wall and are involved in aneurysm development and expansion [33,34,35]. It is thus possible that in these conditions, alterations in collagen organization determine an overall increased risk of concomitant development of IH and AD. In fact, previous studies showed that, in particular, collagen type I may be impaired both in AD and IH. In this view, collagen type I alterations could represent a further common mechanism between AD and IH [36,37]. Furthermore, we found that the risk of AD increased 3.2-fold in current smokers compared with former or non-smokers. Smoking habits have already been found to be a predictor of decreased collagen levels across the transversalis fascia of inguinal hernia [38]. Smoking habits also increase cardiovascular risk by impairing endothelial dysfunction, oxidative stress and nitric oxide availability [39]. We can hypothesize that this behavioral habit may increase the risk for both conditions, namely arterial disease and hernias. Regarding age, it has been shown that in elderly subjects their fibroblasts reduce collagen production, hence favoring hernia development [40]. At the same time, age is an independent cardiovascular risk factor, accelerating the processes of atherosclerosis and endothelial dysfunction [41].

### 4.5. Strengths and Limitations of the Study

This study has strengths and limitations. As for its strength, we may recognize the novelty of the aim of the study. As for its limitations, the single-center design of the study limits the generalizability of the results. Our sample was characterized by an asymmetric gender distribution, with males being more represented than females in all risk categories. Hence, estimates are hardly applicable to females and future studies are needed for this purpose. The lack of a matched control group of patients without IH and with a variable prevalence of AD limits the strength of our findings. Moreover, due to its cross-sectional structure this study is not able to answer the question as to whether one event (e.g., HI, AD or increase in ACR) precedes or follows the other one. However, cross-sectional studies have been designed with the specific aim of generating new hypotheses [42]. As a further limitation, we did not directly measure MMPs in our patients, thus confirming the hypothesis-generating (rather than testing) nature of our study. 

## 5. Conclusions

In conclusion, this study showed a high prevalence of AD in patients with inguinal hernia. Independent risk factors of AD in this setting of patients were old age, increase in ACR and a smoking habit. Based on these findings, a stricter monitoring of AD in patients with IH should be recommended in order to anticipate the prevention of severe cardiovascular events. Similarly, preventive measures against the development of IH should be started in patients with AD, given the association between AD and IH. However, future and hopefully larger studies are needed to confirm our findings. 

## Figures and Tables

**Table 1:** Characteristics of the study patients stratified by arterial disease type.

	No Arterial Disease (*n* = 40)	Carotid Stenosis (*n* = 11)	AAA ^1^ (*n* = 14)	PAD ^1^ (*n* = 5)	*P_adj_*
Age, *years*	55.3 ± 4.9	66.8 ± 5.3	65.3 ± 5.1	65.2 ± 6.3	0.015
Male gender, %	74.9	72.7	78.6	80.0	0.183
Current smokers, %	35.0	36.4	57.1	40.0	0.001
History of cardiovascular disease, %	35.0	36.4	35.7	40.0	0.423
Diabetes, %	25.0	27.3	28.6	20.0	0.612
Hypertension, %	52.5	72.7	64.2	60.0	0.001
Body mass index, kg/m^2^	27.2 ± 3.8	27.5 ± 4.2	27.2 ± 3.9	27.8 ± 5.5	0.825
eGFR, mL/min/1.73 m^2^	72.2 ± 8.3	62.0 ± 6.4	57.5 ± 5.3	50.1 ± 5.0	0.042
Albumin-to-creatinine ratio, mg/g	45 (23–65)	125 (95–180)	215 (125–300)	180 (100–255)	<0.001
Uric acid	5.58 ± 1.45	5.82 ± 1.88	5.59 ± 1.55	6.10 ± 1.73	0.600
LDL cholesterol, mg/dL	119 ± 40	119 ± 40	119 ± 40	119 ± 40	0.392

^1^ AAA, abdominal aortic aneurysm; PAD, peripheral artery disease.

**Table 2:** Particular characteristics of each artery disease category.

	Carotid Stenosis (*n* = 11)
Percentage of stenosis, mean ± standard deviation	57.27 ± 6.07
	Abdominal aortic aneurysm (*n* = 14)
AAA extension in diameter, cm (mean ± standard deviation)	5 ± 1
	Peripheral artery disease (*n* = 5)
Rutherford stage, %	
Stage 1	40
Stage 2	20
Stage 3	20
Stage 4	20

**Table 3:** Correlates for the presence of artery disease in patients with hernias.

Variables	Odds Ratio (95% CI)	*P_adj_*	Variance Inflation Factor
Age, 1 year	1.25 (1.12–3.05)	0.001	1.125
Current smokers, yes vs. no	3.20 (1.65–5.44)	0.001	2.302
Albumin-to-creatinine ratio, 1 mg increase	1.14 (1.05–2.20)	<0.001	0.145

## Data Availability

The data presented in this study are available on request from the corresponding author. The data are not publicly available because an electronic link to the data has not been created.
